# The experience of chronic pain among adolescents: suffering and attempt to overcome pain?

**DOI:** 10.1186/s12887-022-03617-3

**Published:** 2022-09-20

**Authors:** Maryam Shaygan, Azita Jaberi

**Affiliations:** 1 Community Based Psychiatric Care Research Center, Nursing Department, Shiraz University of Medical Sciences, Shiraz, Iran; 2 Community Based Psychiatric Care Research Center, Shiraz University of Medical Sciences, Shiraz, Iran

**Keywords:** Chronic pain, Adolescents, Qualitative study

## Abstract

**Background:**

Chronic pain (CP) among adolescents has received less attention than adultsandthere is limited qualitative studies about it in Iran. This study explored the experience of CP among adolescents.

**Methods:**

This exploratory qualitative study was conducted in April–October 2019. Participants were 14 adolescent students purposively recruited from schools in Shiraz, Iran. Semi-structured interviews were conducted for data collection and data analysis was done through conventional content analysis.

**Findings:**

Adolescents’ experiences of CP came into nine subcategories and three main categories, namely perceived suffering, attempt to overcome pain, and attempt to find sources of support.

**Conclusion:**

Adolescents with CP experience physical and mental suffering and attempt to manage their pain and its associated suffering through different physical and psychological strategies and using different sources of support such as family, peers, healthcare providers, and school staff.

## Introduction

Pain is an unpleasant sensory or emotional experience [1] which is broadly categorized according to its duration as acute or chronic. According to the International Association for the Study of Pain, chronic pain (CP) is a pain which lasts more than 3 months [2]. With a prevalence of 55.7% among American adults, pain is one of the most prevalent health-related problems and one of the leading causes of morbidity in the United States [3]. A study into the eighteen-year trends of pain in the United States reported that the prevalence of pain among American adults increased from 32.9% in 1997–1998 to 41.0% in 2013–2014 [4].

Children and adolescents (CADs) also experience varying levels of different types of pain, including CP [5]. Some associated factors of CP among CADs include critical life events, chronic stress, lack of time for rest and recreation, high levels of activities and homework, and bullying [6, 7]. Despite population-based studies in North America and Europe, because of different assessment methods and time periods measured to assess prevalence of chronic pain in youth, a wide range of pain prevalence was reported [8]. According to these studies, headache is one of the most common pain complaints in children and adolescents [9]. In a systematic review by King et al. (2011) the prevalence of headache ranged from 8 to 83%; abdominal pain from 4 to 53%; back pain from 14 to 24%; musculoskeletal pain from 4 to 40%; multiple pains from 4 to 49%; and other pains from 5 to 88% [10]. Kamper et al. (2016) confirms the wide range for reported prevalence of back pain of 3–39% [11]; and in another meta- analysis, the worldwide prevalence of functional abdominal pain disorders was 13.5% [12].

There has been a large number of publications and investigations on pediatric chronic pain. In addition to many publications on chronic pain in the pediatric population between 2000 and 2022, there are also a number of texts dedicated to pediatric chronic pain. These studies depicted that pain has dramatic effects on adolescents and their families. CADs with CP have frequent absences from school [13, 14], miss social activities, and experience sleep disorders, low satisfaction with life and overall well-being [15], emotional problems [16] and other physical problems later in life [17].

Decreasing the prevalence of chronic pain in children and adolescents demands to increase efforts in a better understanding of children’s pain experience and how they deal with it. Effective implementation of this knowledge translation entails a strong basis of evidence and a positive context where evidence is implemented (e.g., culture) [8]. Nonetheless, the top most existing articles consisted of randomized controlled trials and epidemiological studies, especially in acute pain [18]; therefore, a paradigm shift to paying attention to prolonged and persistent pain is essential [8]. Meanwhile, since previous personal experiences of pain [19], cognitive awareness, cultural and educational factors, developmental conditions, and pain characteristics [14] may make pain experience unique to each person, qualitative research should be considered to better understand the experiences of youth with chronic pain [8].

Hence, there isn’t much qualitative research, and most of them are on an ethnically and culturally narrow sample (i.e. European and North American, generally Western, Educated, Industrialized, Rich, Democratic countries). One of the studies in this area reported that the three main themes of adolescents’ concerns about CP were worry ripple, pain mystery, and avoidance from conundrum. That study mainly focused on fears and concerns and did not address other aspects of CP experience among adolescents [20]. Another study qualitatively explored pain management and attitudes towards using over-the-counter analgesics among adolescents with CP and reported that they considered CP unmanageable and unavoidable. That study also did not provide in-depth data about the different aspects of adolescents’ experiences of CP [21]. Moreover, a study into the development of a web- and mobile-based self-management program reported that there were limited data about the CP-related cognitive and emotional experiences of adolescents with CP [22]. In other qualitative studies adolescents describe pain as interrupting their ability to be “normal” and making them feel different from their healthy friends [23], while medical providers, school personnel, peers and even family members enact pain-related stigma toward adolescents [24].

Considering the paucity of literature related to the CADs regarding CP experience in Iran and because management of chronic pain requires a holistic approach which incorporates the experienced psychological and social consequences of pain by the adolescents, there is a need for new appropriate interventions for effective CP management based on the viewpoints of the patients. Uncertainties and limited in-depth data about the different aspects of CP experience among adolescents highlight the importance of further studies in this area. This study was conducted to narrow this knowledge gap. The aim of the study was to explore the experience of CP among adolescents, and specific objectives were “CP management”, “CP-related emotions and thoughts”, and “expectations during CP experience”.

## Methods

### Design

This exploratory qualitative study was conducted in April–October 2019. We adopted a qualitative research approach to explore adolescents’ perceptions, feelings and experiences of chronic pain, by conducting in-depth interviews. We purposefully chose four different junior high schools from two different district with different socio-economic status, two schools were girls and other two schools were boys, in order to broaden the range of opinions. Because of the paucity of data in this area, it was essential to first explore how adolescents view chronic pain; this was best facilitated within a methodology yielding the greatest depth and breadth of information.

### Participants and setting

Participants were 7th to 12^th^grade students from four junior high schools in Shiraz, Southwest of Iran. Approval for the study was obtained from ethics review board of Shiraz University of Shiraz, school principals and teachers. One week before the study began, consent forms describing the study were distributed to students; parental and adolescents’ were obtained. Participants were recruited directly or telephone numbers were obtained and they were subsequently telephoned. All participants that were contacted, accepted to participate in the study, so there were not potential participants who were approached but did not consent.

Participants were 14 adolescents purposively recruited from the study setting with maximum variation respecting their age, gender, educational level, and duration of CP. Purposive sampling was conducted to ensure selected participants had different socio-demographic characteristics. Inclusion criteria were an age of 12–18 years, affliction by CP based on the criteria provided in the 11th revision of the International Classification of Diseases, agreement for participation in the study, ability to provide rich data about the study subject matter, physical health and ability (for example, no hearing or speech problems such as stuttering), and mental health to answer the interviewer’s questions. In order to obtain the fullest range of pain experiences, exclusion criteria were restricted to only the inability read or speak Persian and developmental disability. The diagnosis of chronic pain, defined as pain that is described as chronic, persistent or recurrent pain with a minimum duration of at least 3 months, was verified by the first author (who is an expert in chronic pain) based on three screening questions according to the 11th revision of the International Classification of Diseases (ICD-11) [25]: 1) Do you currently have pain, and if so is it permanent or intermittent? 2) Have you experienced this pain or discomfort for more than 3 months? 3) Does this pain and discomfort affect your life and activities?

### Data collection

Data were collected by conducting semi-structured face-to-face interviews. An interview guide was developed for interviews through consulting school teachers, nurses, psychiatric nurses, pain psychologists, and pain specialists (e.g., physicians). The guide was piloted by the second author in a single interview and then, was revised by the first and the second authors. The main topics of the interviews were CP management, CP-related emotions and thoughts, and expectations during CP experience. Examples of interview questions were, “How do you cope with your pain when you have pain?” “Which strategies do you use to reduce your pain?” “What do you think about when you have pain?” “How do you feel when you have pain?” and “What expectations do you have when you have pain?” Interviews were started using broad questions and were continued using follow-up clarifying questions such as “Can you provide more explanations about this?“Interviews took place between April and August 2019, conducted in Persian language, with probing as needed. All interviews were conducted in a private room by the second author who was experienced in qualitative research. The duration of the interviews was 30–45 minutes. Sampling and data collection were continued up to data saturation. All interviews were audio-recorded and later transcribed.

### Data analysis

Data were analyzed concurrently with data collection using conventional content analysis proposed by Elo and Kyngäs (2008) [26]. Concurrent data collection and analysis facilitated access to key informants and helped develop and use better questions for the next interviews. Initially, the content of each interview was transcribed word by word and the transcript was perused and coded. Relevant codes were grouped into subcategories and relevant subcategories were grouped into larger categories. The labels of the subcategories and categories were flexible and were revised during data analysis. Both authors got involved in data analysis. Data were organized and managed using the MAXQDA software 2007.

### Rigor

The common criteria for applying rigor to qualitative studies were used in this study [27]. Credibility was ensured via prolonged engagement with the study (for 7 months), immersion in the data, peer checking by experts in qualitative studies and CP, and member checking. Transferability was ensured through providing detailed information about the study and its findings so that other researchers in the area of CP among adolescents can use the findings of the study as a source of information. Moreover, dependability was ensured via an audit trail with detailed descriptions about data collection, interview guide, data analysis, and participants’ quotes.

### Ethical considerations

The Ethics Committee of Shiraz University of Medical Sciences, Shiraz, Iran, approved this study (code: IR.SUMS.REC.1398.1099). Permission for data collection was obtained from participants’ parents and the authorities of the study setting. Moreover, informed consent was obtained from participants and their parents. Participation in the study was voluntary and participants were able to withdraw from the study at will. Interviews were conducted with appointment and participants were ensured of confidential data management.

## Findings

Participants were seven male and seven female adolescents with a mean age of 14.5 ± 1.95 years. Table 1 shows their characteristics.

**Table 1:** Participants’ characteristics

No.	Age (Years)	Gender	Educational year
p1	13	Female	6
p2	13	Female	6
p3	14	Female	7
p4	12	Female	5
p5	13	Female	6
p6	13	Female	6
p7	14	Female	7
p8	15	Female	8
p9	17	Male	10
p10	16	Male	9
p11	17	Male	10
p12	12	Male	5
p13	17	Male	10
p14	17	Female	10

Participants’ experiences of CP were categorized into three main categories, namely perceived suffering, attempt to conquer pain, and hopes for support (Table 2).

**Table 2:** The categories, subcategories, and examples of the codes of the study

Codes	Subcategories	Main categories
Thought patterns; Grief and sorrow; Reduced self-confidence; Self-blame; Feeling of guilt	Internal reactions to pain	Perceived suffering
Aggression; Crying	External/Observed reactions to pain	
Gender; Family education; Subjective burden of pain.	Factors affecting pain management	Attempt to overcome pain
Medical therapies; Traditional and herbal medicine therapies	Physical strategies for pain management	
Avoidance or distraction; Resilience; Optimism	Psychological strategies for pain management	
Parents; Siblings	Family support	Hopes for support
Peers; Classmates	Peer support	
School staff; Teachers	Social support	
–	Spiritual management	

### Perceived suffering

One of the experiences of adolescents with CP was perceived suffering due to CP. CP and its long-term course were associated with a suffering which might beinvisible and imperceptible toothers. Participants showed their perceived suffering through internal and external reactions.

#### Internal reactions to pain

Participants had some internal reactions to pain which were invisible to others. One of these reactions was thought patterns regarding the pain that might affect the perception of pain. These negative or positive thought patterns about pain could increase the perceived suffering of pain or eliminate it. Some participants mentioned negative thought patterns as follows:

*I had terrible thoughts about my pain (P. 9)… I had some concerns ... for example I thought it might be cancer (P. 1). I was tired of it … (P. 2) I was telling myself that I cannot be healed anymore (P. 4) Why did this happen to me. It might be my fault (P. 6).*

On the other hand, there was a participant that was trying to put this suffering behind with positive thought patterns.

*I told myself that this was not a big deal and it would be fine soon… it was not good if I became isolated … because it might make me depressed and stressed and anxious, like it had happened in my grandfather before (P. 3).*

Other internal reactions were sorrow and grief and were common, particularly among female participants.

*I usually have sadness and anger. I get tired and upset when I have pain. Of course, it depends on pain intensity and may even have outward manifestation (P. 2).*

Another internal reaction to CP among participants was their concern with rejection by friends and classmates.

*I have fear over being unable to be with my friends and fear that they distance from me, blame me, and say that I exaggerate my problem (P. 5).*

Some participants expressed their internal reactions and symptoms and noted that the solution to such problems was to use counseling services provided inside and outside the school.

*I felt upset due to pain; but, if someone gave me counseling, I became hopeful and controlled my pain (P 5).*

#### External/observable reactions to pain

Participants noted that when pain lasted too long and treatments were ineffective, they were likely to show behaviors such as aggression. This can be considered as a very likely reaction, particularly due to hormonal changes during adolescence.

*When I have pain, I become aggressive because I run out of patience and take my anger out on others. A person who has been able to easily perform his/her daily activities but is no longer able to do so will easily get upset and may even get angry (P. 6).*

*[When I have pain,] I become sensitive and get frantic. I easily get frantic and then, become calm very soon (P. 7).*

Crying was another external reaction to suffering, particularly among female participants. They attributed this reaction to the lack of perceived empathy. In other words, they might not show such reactions if they felt that a person from family, friends, or school staff understood their conditions.

*Sometimes, I cried but it was not due to pain itself; rather, I wished there was somebody who helped and understood me (P. 4).*

However, from the perspective of some participants, crying might has happened as a result of some other factors, i.e. chores that girls might have in the house or the differences between boys and girls, that is, girls cry more often in facing the life challenges:

*Girls have more challenging situation at home because they have more chores than boys … that’s why the boys are less agitated and restless (regarding chronic pain) … instead the girls are crying more (in this kind of situations) (P. 11).*

#### Factors affecting pain management

Participants’ experiences showed that a series of factors affect their choice and use of pain management strategies. These factors were gender, family education, and the subjective burden of pain.

##### Gender

Some participants believed that pain management strategies among girls are different from those among boys and noted that adolescents may choose physical or psychological strategies for pain management according to their gender. They also noted that reaction to pain also depends on gender so that girls usually have lower pain threshold. In this regard, two girls quoted:

*Women have different personality traits. They sometimes express their problems and sometimes avoid such expression. This is also true for men; but men mostly avoid expressing their pain unless they cannot manage their emotions (P. 6).*

*Women have more difficult conditions because they have greater responsibility at home. On the other hand, men have higher resistance. Restlessness is less common among men and more common among women. Women cry (P. 7).*

Some female participants noted that men and women differ from each other respecting their internal and external reactions to pain.

*Boys are more resistant to emotional pain, while girls are more resistant to physical pain. In response to physical pain, boys are impatient and aggressive, while girls are calm but may get sad or depressed (P. 2).*

##### Family education

Participants noted that family education and environment have significant role in pain tolerance and management. They highlighted that parents’ responses to pain can be a model for adolescents. They also believed that birth rank can affect adolescents’ pain perception and experience.

*A child with a father who well tolerates pain will have greater pain tolerance. Moreover, I have greater pain tolerance to pain compared with my little brother because he has been reared in a way that shows lower tolerance (P. 9).*

*Family is very important. A person who has worked and experienced difficulties since childhood has greater tolerance (P. 7).*

##### The subjective burden of pain

Participants noted that subjective perception and image of pain can affect pain tolerance.

*In my opinion, the subjective burden of pain is more important than the pain itself. Therefore, we should tell a patient that he/she can overcome pain (P. 2).*

### Attempt to overcome pain

The second main category of the study was attempt to conquer pain. Although participants were under the heavy burden of pain-related suffering, they had not surrendered to it and sought strategies to conquer it. Some of them referred to their attempt for conquering pain as fighting with disease. While adolescents are usually expected to overcome their problems with others’ help, study participants were able to use appropriate strategies to independently overcome their pain. These strategies were grouped as physical strategies and psychological strategies for pain management.

#### Physical strategies for pain management

Physical strategies such as analgesics, heat therapy, and topical ointments were among the strategies participants used for managing their pain. Moreover, as traditional and herbal medicine products are widely used in Iran, participants reported the use of these products for pain management based on their parents’ recommendations. Some of these products included different herbal teas and topical use of pepper.

*First, we use medicines which are found at home like analgesics. For example, we use flixweed and herbal teas such as thyme for abdominal pain or use pepper or heat for muscle pain (P. 2).*

Almost all participants noted that they referred to doctor if home therapies were ineffective. In other words, a physical strategy for pain management used by participants was to seek medical advice.

*We refer to doctor when the drug administered by my family or doctordoes not help (P. 8).*

#### Psychological strategies for pain management

The second subcategory of the attempt to conquer pain main category was psychological strategies. Participants attempted to conquer their pain using psychological methods which they had implicitly learned from their parents. In other words, they used strategies they had seen their parents used for managing their own pain. These strategies were avoidance or distraction, resilience, and optimism.

##### Avoidance or distraction

Participants attempted to deviate their attention from their pain and improve their endurance through avoidance or distraction. Accordingly, they attempted not to think about their pain or attempted to think about other things.

*We shouldn’t think about pain; rather, we should think about other things such as future. Negative thoughts and autosuggestion postpone our recovery and increase our pain perception (P. 2).*

They also resorted to activities which helped them avoid focusing on their pain. Examples of these activities were listening to music, studying, watching television, communication with friends and family members, and talking to others.

*I engage in entertaining activities. For example, I communicate with my friends. If I become alone, I feel depressed and will have worse feelings (P. 5).*

*In these situations, I prefer to go on a travel, visit my friends, or go to cinema (P. 7).*

Participants also attempted to deviate their attention from pain through engaging in artistic activities, gardening, or keeping pets.

*I do my favorite activities such as growing flowers and plants, artistic activities, keeping birds, walking in the park, or feeding animals. These activities give me energy (P. 5).*

##### Resilience

Some participants noted that when facing CP, they attempted to cope with it and keep patience. They considered their pain as a trivial thing and attempted to tolerate it. Although resilience is a teachable strategy for coping with stressors and crises, our participants used it to cope with their CP without receiving any education about it and knowing its scientific underpinnings. They attempted to reduce the negative effects of CP on themselves instead of having negative thoughts about it such as suicide or possibility of developing cancer.

*I have horrible thoughts about my illness. For example, I think I have gotten cancer. Nonetheless, I seek solution (P. 6).*

*Of course, my pain aggravates or lasts too long. But, I keep patience and cope with it (P. 3).*

##### Optimism

Some participants attempted to have positive thoughts. They believed that their pain would not be permanent and would finish 1 day. They attempted to boost their hope through this optimistic approach to pain.

*When we have pain, we should think about positive things such as the fact that we can continue our life (P. 5).*

*We should avoid stress, anxiety, and negative thoughts (P. 4).*

### Hopes for support

The third main category of adolescents’ experiences of CP was their hopes for support. Besides physical and psychological strategies which they used for pain management, they expected to find sources of support such as family, peers, social sources, and spiritual methods.

#### Family support

One of the basic sources of support for adolescents with CP was family members, particularly parents. Participants noted that they liked to have their parents’ kindness when they experienced stress and worry. They also expected their families to provide them with empathy, sympathy, help in using physical strategies for pain management, and help in referring to doctor.

*I expect my parents to understand me, do not leave me alone, give me hope, and fulfill my requests (P. 4).*

Female participants reported that they preferred to be supported by their mothers. They noted that techniques such as verbal communication, story telling, and talking about good memories by their mothers are indicative of their mothers’ kindness toward them.

*I like my mother to talk to me or tell me stories because in this way, I forget my pain (P. 4).*

*I like them to remind me of the memories of good days (P. 5).*

According to the participants, other family members, such as siblings, also have significant role in supporting adolescents with CP. They can reduce CP-related suffering through talking to adolescents, playing with them, and boosting their hope.

*My siblings can play important role in giving hope to me [for example] through playing with me (P. 8).*

Of course, some parents and siblings did their sympathy support:

*My pain was eliminating when I was talking to my sister (P. 4).*

*My mother was fondling me and giving me a massage in the pain site (P. 7).*

Significant others, particularly school staff, can also play significant role in supporting adolescents who suffer from CP, which in the some cases, these helps were provided by the school authorities, specially the teachers.

*I expect my significant others to support me, help me, and boost my morale. [For example,] I expect my teachers not to give me heavy assignments and give me more rest opportunities (P. 3).*

*Our teacher let my friend to write my notes in the class or at examination time (he let someone to write my answers while I was dictating to that person) (P. 11).*

#### Peer support

Peers were another source of support from the perspectives of participants. They noted that due to CP-related limitations, sometimes their classmates and peers should help them. For instance, they expected their classmates and peers to help them in taking their belongings from classroom to school yard, provide them with psychological support, and avoid isolating or ridiculing them.

*I attempt to communicate with my friends when I have pain (P. 4).*

*I expect my friends not to talk about me behind my back, not to reject me, not to accuse me of pain exaggeration, and not to avoid playing with me for my sickness (P. 12).*

One participant mentioned that this sympathy was taken from their classmates and friends.

*My friends took care of me with consideration and there were no jokes about my condition (P. 9).*

#### Spiritual management

Some participants preferred resorting to spiritual or religious rituals when they had pain. For example, they recited the Holy Quran or read stories about Imams. As children in Iran grow in a religious background, they usually resort to spiritual and religious sources when they face crises or stressors.

*For example, I like story telling. I like my auntie to recite Quran for me or tell stories about Imams (P. 2).*

## Discussion

This study aimed at exploring the experience of CP among adolescents. Findings showed that adolescents with CP experienced different CP-related suffering including internal and external reactions, but they attempted to conquer CP and minimize its effects through using different physical and psychological strategies while they were hoping for supports.

Participants of the study suffered from pain and reacted to that by some internal processes. The study findings emphasized the role of cognitions in adolescents with chronic pain in the subcategory of “thought patterns”. According to the literature, the developmental process of children and adolescents leads to gradual changes in their emotional and cognitive processes in various fields. Consequently, cognitive dimensions related to pain, such as selective attention, interpretation of ambiguity and selective memory emerge after the evolutionary period. For example, choosing an interpretation in a situation like chronic pain happens when the child has earned abstract thinking, so he/she can interpret pain as a not so terrible phenomenon, or he/she can express appropriate responses using selective memory. The use of strategies to divert attention from chronic pain is also created when the mature attentional-shifting have been formed [28]. In this way, the opinions of the participants in present study may include cognitive biases, some of which are related to the developmental process of children and adolescents. Therefore, conducting longitudinal studies using direct and indirect methods of measuring these cognitive processes can provide more evidence regarding this aspect of chronic pain and its relationship with cognitive processes in children and adolescents. Hence, the current results may present the thinking patterns and specify the way Iranian youth think about painwhich subsequently might help the therapists to develop appropriate culturally sensitive CBT treatments.

Study findings showed that adolescents with CP experienced different symptoms and problems due to CP such as grief, sorrow, reduced self-confidence, and fear over rejection by peers. In line with these findings, several earlier studies showed psychological problems as the consequences of CP [20, 21] and a study showed psychological disorders both as a contributing factor and a consequence of CP [29]. Several other studies reported the significant relationship of CP with quality of life [30], mental well-being, social isolation [31], fear over future, fear over others’ perceptions, and fear over others’ verbal reactions to CP [32]. All these findings imply that emotional distress is an important aspect of life among adolescents with CP. Therefore, employing effective strategies to manage emotional distress in their daily life is of great importance.

We also found fear over rejection by peers as a main concern of adolescents with CP. Children with CP in another study reported that they felt their peers misunderstood them and noted that they needed greater attention and better understanding by others when they experienced severe pain [33]. Studies on adolescents with chronic conditions such as chronic kidney disease and sickle cell anemia also reported negative feelings toward being different from others as one of their main concerns [34, 35]. CADs learn through environmental mirror how to regulate their feelings and physiological responses. Therefore, peers’ and friends’ reactions may affect their emotions so that they may feel social stigmatization due to pain and decide just to express their pain and hide their pain-related emotions such as anxiety or anger [36]. These findings highlight the importance of public education about CP in order to prevent a sense of stigmatization among adolescents with CP.

Participants in the present study attempted to use different physical and psychological strategies to manage their CP and its associated physical and mental problems such as negative thoughts and loneliness. In a former study, adolescents who considered their pain manageable used problem-oriented strategies to manage their pain, while those who considered their pain as communicable used remedies at hand and more frequently expressed their emotions [21]. In the present study, this difference was gender-based, so that the participants expressed stereotyped gender narratives. This finding may be due to gender-related beliefs in the Iranian context which expect boys to be more resistant to problems than girls.

Our participants’ attempt to manage their CP using physical and psychological strategies can be considered as an attempt for self-management. By definition, self-management is to feel responsibility towards the symptoms and the treatments of a chronic condition and includes coping with its related physical, mental, and cognitive changes and adoption of an appropriate lifestyle based on the existing conditions. This definition highlights that self-management relies on acquiring information, learning about behavioral modification, and promoting self-confidence [37]. Acquisition of adequate knowledge and skills is a key component of effective self-management among patients with chronic conditions. Therefore, adolescents with chronic conditions such as CP should be provided with quality education and informational support regarding self-management in order to empower them for identifying their needs and using appropriate strategies to fulfill them [35, 38].

Our findings also showed that adolescents with CP used strategies such as resilience, avoidance, and optimism for managing their CP. A study on adolescents with chronic conditions such as irritable bowel disease also reported that they used internal coping strategies for coping with their conditions [39]. These findings highlight that quality education and counseling may help adolescents use effective strategies for CP management. As the present study did not explore adolescents’ experiences over time, longitudinal studies on adolescents with CP may provide more detailed information about their coping strategies. Our findings also revealed that adolescents with CP used spiritual methods for pain management. This finding may be due to the prevalence of religious and spiritual beliefs in the Iranian context [40]. To the best of our knowledge, none of the previous studies reported this finding.

We also found that participants’ strategies for pain management were affected by gender, family education, and the subjective burden of pain. Similarly, a former study reported that contextual and illness-specific factors were linked to general performance and quality of life among adolescents with CP [39]. The social context, such as the parent– child relationship, in understanding sex differences in children pain, are believed to be learned in social environments such as children families. For example, a family that stands for traditional gender roles may respond to their daughters’ pain with empathy and support, whilst they might respond to their sons’ pain with this message that he is expected to be strong in response to his pain [8].

An important finding of the present study was adolescents’ attempt to find sources of support for the better management of their pain. These sources were family members, peers, and school staff. Adolescents with sickle cell anemia in a former study also referred to self-management as a joint effort, implying that different individuals and groups need to get involved in the management of chronic conditions among adolescents [34]. Another study reported that the social environment of children, particularly family members and peers, has significant role in the development of pain disorders [41]. Moreover, a study proposed models for explaining family relationships in CP which highlighted that CP perception among CADs affects and is affected by family [42]. Studies for assessing the effects of joint parent-child strategies on CP perception among CADs are needed to produce clearer evidence in this area. The experience of one of our participants showed that parents’ approach to pain can affect adolescents’ pain experience. CP assessment models also consider the role of parental reinforcement and modeling in pain experience among CADs. While there is few qualitative studies in the area, Umberger et al. have identified positive impacts of parental pain, such as children’s increased independence and development of empathy [43]. Further studies in this area are recommended.

Study findings showed that adolescents with CP attempted to find peer support in order to cope with their pain. Peer support plays important role in managing problems associated with chronic conditions among CADs aged 5–18 years [33, 39]. A study also reported school environment as a good environment with potentials for effective pain management [44]. These findings denote that peer support can be considered as a strategy for effective CP management among adolescents. Nonetheless, adolescents with CP may experience disruption in their relationships with their peers. A systematic review concluded that CADs with CP usually have fewer friends and are more isolated [45]. Therefore, healthcare providers and school staff need to work together in order to attract stronger peer support for adolescents with CP [46].

To the best of our knowledge, this was a novel study into the experiences of CP among adolescents in an Asian community. Other strengths of this qualitative study include attention to the Iranian pediatric chronic pain, recruitment of participants from different schools which could potentially explore between different environments/cultures, inclusion of both girls’ and boys’ schools. A further strength was the use of open ended questions so that a variety of themes could emerge. The qualitative data obtained from the present study can be used for experimental designs investigating the impact of strategies on adolescents’ pain behaviors, attitudes, and expression among adolescents with CP.

However, the study had some limitations. First, the findings may not be representative of the whole children and adolescents considering that this study took place in the schools of one province of Iran; however, since the purpose of a qualitative study is to explore the social reality of a particular group at a particular time, generalization or transferability is not the ultimate goal. Second, there might be selection bias because those selected were based on their availability and willingness to participate. Furthermore, the study solely focused on adolescents’ experiences and did not explore parents’ and school staff’s experiences. Moreover, data collection was performed using a single technique, i.e., semi-structured interview. Future studies in this area are recommended to use data triangulation in order to collect more in-depth data. Additionally, details regarding the adolescents’ pain experiences such as duration of chronic pain, location, frequency and level of disability (e.g., school absenteeism) were not obtained; therefore, considering them in future studies are warranted.

## Conclusion

This study shows that adolescents with CP attempt to manage their pain and its associated suffering through different physical and psychological strategies and using different sources of support such as family, peers, healthcare providers, and school staff. These findings might extend our knowledge of adolescent CP experiences in an environment where cultures and social practices may differ from North America and Europe.

The clinical implications for the further development of this research include a better understanding of the aforementioned factors that impact how adolescents interpret and express pain and using that knowledge to provide better supportive resources for adolescents with CP. Healthcare providers can use the findings of the present study to develop strategies for better CP management among adolescents. These strategies may include community-based educations, particularly for school staff and family members, age-appropriate and holistic multidisciplinary services for afflicted adolescents, and web- and mobile-based educations about pain management techniques such as relaxation techniques and mindfulness. Future studies are recommended to test biopsychosocial and family-centered models of pain management in the Iranian culture. Although these models were developed in cultures different from the Iranian culture, they include important aspects of CP management which may help prevent and manage CP among adolescents.

## Data Availability

The datasets used and/or analyzed during the current study are available from the corresponding author on reasonable request.

## Abbreviations

CP: Chronic Pain
CADs: Children and adolescents
